# Ruthenium(II) Complexes with 2-Phenylimidazo[4,5-f][1,10]phenanthroline Derivatives that Strongly Combat Cisplatin-Resistant Tumor Cells

**DOI:** 10.1038/srep19449

**Published:** 2016-01-14

**Authors:** Leli Zeng, Yu Chen, Jiangping Liu, Huaiyi Huang, Ruilin Guan, Liangnian Ji, Hui Chao

**Affiliations:** 1 MOE Key Laboratory of Bioinorganic and Synthetic Chemistry, School of Chemistry and Chemical Engineering, Sun Yat-Sen University, Guangzhou, 510275, China

## Abstract

Cisplatin was the first metal-based therapeutic agent approved for the treatment of human cancers, but its clinical activity is greatly limited by tumor drug resistance. This work utilized the parent complex [Ru(phen)_2_(PIP)]^2+^ (**1**) to develop three Ru(II) complexes (**2**–**4)** with different positional modifications. These compounds exhibited similar or superior cytotoxicities compared to cisplatin in HeLa, A549 and multidrug-resistant (A549R) tumor cell lines. Complex **4**, the most potent member of the series, was highly active against A549R cancer cells (IC_50_ = 0.8 μM). This complex exhibited 178-fold better activity than cisplatin (IC_50_ = 142.5 μM) in A549R cells. 3D multicellular A549R tumor spheroids were also used to confirm the high proliferative and cytotoxic activity of complex **4**. Complex **4** had the greatest cellular uptake and had a tendency to accumulate in the mitochondria of A549R cells. Further mechanistic studies showed that complex **4** induced A549R cell apoptosis via inhibition of thioredoxin reductase (TrxR), elevated intracellular ROS levels, mitochondrial dysfunction and cell cycle arrest, making it an outstanding candidate for overcoming cisplatin resistance.

Cisplatin is an effective antitumor agent that acts on DNA and is largely employed as the first metal-based therapeutic in the clinic against a wide spectrum of solid tumors^1^,^2^. However, drug resistance to cisplatin limits its applications and represents a continuing challenge^3^. Drug resistance mainly arises from different cellular adaptations, including reduced cellular drug concentration, increased rates of drug damage repair and drug deactivation^4^. Theoretically, there is a need for an effective anticancer drug that exhibits increased cellular uptake in tumor cells and is able to maintain sufficient drug concentrations to kill cancer cells^5^,^6^. Compared with platinum agents, some of the new transition metal complexes breakdown less easily, which is an important property for the delivery of drugs to locations where they are needed to fight cancers in the body^7^,^8^. Worldwide efforts to develop alternative organometallic drug designs that are distinct from cisplatin and have different targets have been directed toward overcoming this issue^9^,^10^,^11^,^12^,^13^,^14^.

Due to their octahedral geometry, ruthenium complexes are widely utilized to construct highly effective anticancer agents with high selectivity and fewer (and less severe) side effects compared to platinum drugs^15^. Ruthenium complexes have been investigated for use as DNA topoisomerase inhibitors^16^, TrxR inhibitors^17^, antimicrobial agents^18^, molecular probes^19^, and anticancer agents^20^. Gratifyingly, three ruthenium-based chemotherapeutics are currently in clinical trials. Some ruthenium complexes have been proven to be mitochondria-targeting anticancer drug candidates^21^, which often induce redox reactions inside cancer cells resulting in an increase in reactive oxygen species (ROS)^22^. Some studies have observed reduced mitochondrial accumulation of cisplatin in cisplatin-resistant cells^23^; in contrast, ruthenium-based drugs have been found to have different subcellular distributions and no decrease in the amount of ruthenium was observed in cisplatin-resistant cells^24^. Moreover, complexes with mitochondria-targeting functionality have been created as efficient anticancer drugs that are immune to cisplatin resistance^25^,^26^. Therefore, mitochondria-targeting Ru(II) complexes are potential strong candidates for combating cisplatin-resistant tumor cells.

Fluorine substituents have become a common and important drug component. They enhance the lipophilicity and biological activity of drug compounds^27^,^28^, and their introduction has been facilitated by the development of safe and selective fluorinating compounds^29^. Accordingly, the design of drug-like heterocyclic organic small molecules with trifluoromethyl groups that chelate ruthenium has generated promising anticancer drug candidates^30^. In addition, 2-phenylimidazo[4,5-f][1,10]phenanthroline (PIP) and its derivatives are widely used in medicinal chemistry. Ru(phen)_2_(PIP)^2+^ is a famous mitochondria-targeting Ru(II) complex^31^. As shown in scheme 1, a PIP ligand modified by the incorporation of a trifluoromethyl group into the benzene ring is a core component of our design. Often, 1,10-phenanthroline (phen) is directly used as a bis-chelating ligand to build Ru(II) polypyridyl complexes. The C-N coordination site of the 7,8-benzoquinoline (bq) ligand cyclometalates ruthenium, which can decrease the positive charge of the Ru metal center and increase cellular uptake^32^,^33^,^34^. The hydrogen (H) atom of the NH-functionality in PIP was substituted by a tert-butyl-benzene group to increase lipophilicity. The trifluoromethyl functionality was installed into the PIP ligand as a functional ligand to improve not only the bioavailabilities and membrane permeabilities of the complexes but also the interactions of the Ru complexes with biomolecules. Therefore, we synthesized four Ru(II) complexes with similar structures but distinctly different biological activities to verify that ruthenium cyclometalation in combination with trifluoromethyl and PIP ligands is a simple but competitive method to develop novel metallodrugs for the treatment of cancer.

In this work, we studied the changes in biological activity and physicochemical properties resulting from structural modifications of the four Ru(II) complexes (Fig. 1). Complex **4** successfully exhibited potent *in vitro* cytotoxicity that was higher than cisplatin and the other three Ru(II) complexes against all of the screen cancer cell lines. We established 3D multicellular tumor spheroids based on A549R cells, and used this model to investigate the *in vitro* activity of complex **4** toward multidrug-resistant (A549R) tumor cells. The cellular uptake and localization of complex **4** in A549R cells were studied. Furthermore, we investigated the mechanism of complex **4**-induced A549R cell apoptosis. The results show that complex **4** can efficiently induce A549R cell apoptosis *via* multiple pathways.

## Results

### Syntheses and Characterization

The **tbtfpip** ligand and Ru(II) complexes were characterized by ESI-MS, ^1^H NMR and elemental analyses (Figures S1–S7). The main ligand, 1-(4-tert-butylphenyl)**-**2-(4-(trifluoromethyl)phenyl)imidazo[4,5-f][1,10]phenanthroline (**tbtfpip**), was efficiently synthesized from a mixture of ammonium acetate, 1,10-phenanthroline-5,6-dione, 4-tert-butylaniline and 4-(trifluoromethyl)benzaldehyde in glacial acetic acid under refluxing reaction conditions. The syntheses of complexes **1**^30^ and **2**^35^ have been previously reported, and complex **3** was synthesized using Ru(phen_2_)Cl_2_·2H_2_O and **tbtfpip** according to previously published procedures. Complex **4** was synthesized from the reaction of [(η^6^-C_6_H_6_)RuCl_2_]_2_ with benzo[h]quinoline, 1,10-phenanthroline and **tbtfpip** in 30% yield. Only the monocationic signal of [M-ClO_4_]^+^ was detected in the ESI-MS spectra of complex **4** (Figure S4); however, a bicationic signal of [M-2ClO_4_]^2+^ was also observed for complex **3** (Figure S3). In the ^1^H NMR spectra of complexes **3** and **4** (Figures S6 and S7), the proton resonance ortho to the C_aryl_ atom bound to the Ru center in **4** (6.40 ppm) was shifted upfield by 1.01 ppm compared to the corresponding proton in complex **3** (7.41 ppm). The intrinsic variation of the proton ortho or para to the C_aryl_ atom bound to the Ru center was the main difference between the cyclometalated and non-cyclometalated complexes^36^. In addition, the lipophilicity of these four complexes was measured (Figure S8). Complex **1** had negative log *P*_o/w_ values, suggesting hydrophilicity. Complexes **2** and **3** exhibited moderate log *P*_o/w_ values due to their trifluoromethyl groups, indicating that they were hydrophobic. In contrast, the cyclometalated complex **4** had the largest log *P*_*o/w*_ value and was proven to be the most hydrophobic.

### Cytotoxicity in 2D Cancer Cell Cultures

Compounds **1**–**4** were evaluated against three tumor cell lines derived from different tissues, including the cervix (HeLa), lung (A549) and cisplatin-resistant lung (A549R) tissues. For comparison, the cytotoxicity of cisplatin was also evaluated. As a control, the toxicities of the complexes were also tested against the normal human cell line (LO2). Table 1 shows that the *in vitro* antiproliferative efficacies of the complexes were observed in the following order: **4**> cisplatin >**3** >**2** >**1**. Remarkable differences were observed among the four complexes: the cyclometalated Ru(II) complex **4** exhibited much higher cytotoxicity (IC_50_ values ranged from 0.8 to 2.4 μM) toward all the tested cancer cell lines compared to the other three Ru(II) complexes. This suggested that the trifluoromethyl substitute, the tert-butyl-benzene group and cyclometalation all improved the anticancer activities of the Ru(II) complexes. Notably, complex **4** was less cytotoxic to the LO2 cell line (6.7 μM) than to the cancer cell lines, indicating its selectivity for cancer cells. Most importantly, complex **4** was the most active compound against the A549R cell line and had an IC_50_ value of 0.8 μM compared with 142.5 μM for cisplatin (a 178-fold difference); this indicated that complex **4** could be an active candidate for combating cisplatin-resistant cancers.

**Figure 2:**
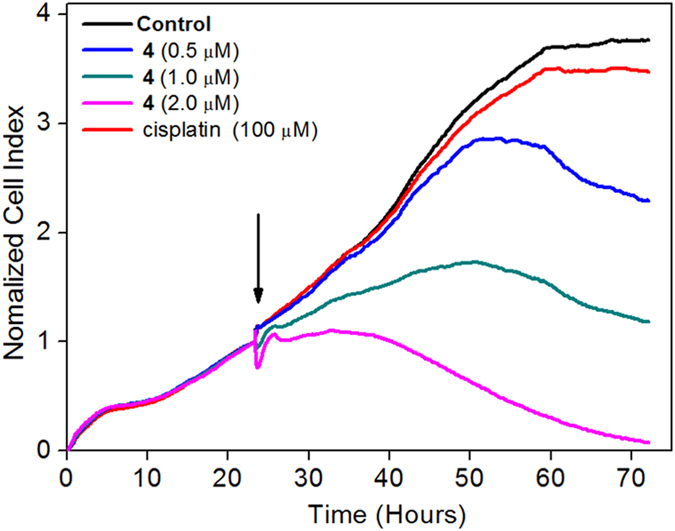
Kinetics of cytotoxicity responses for complex **4** in A549R cells monitored by the xCELLigence system. The arrow signifies the time of complex **4** addition.

In addition, a real-time cell growth and proliferation assay using an xCELLigence System was utilized to confirm the high toxicity of complex **4** on A549R cells^37^. As shown in Fig. 2, cisplatin (100 μM) had a negative effect on A549R cells compared to the control. In contrast, A549R cells were killed by treatment with complex **4** in a concentration-dependent manner. Cells treated with high doses of complex **4** (2.0 μM) experienced rapidly abolished cellular proliferation, indicating that the cells underwent irreversible senescence. On the other hand, cells treated with minimal doses of complex **4** (0.5 μM) began to die after a short delay. These results confirmed the high cytotoxicity of complex **4** against A549R cells.

### Cytotoxicity in a 3D MCTS cancer model

**Figure 3:**
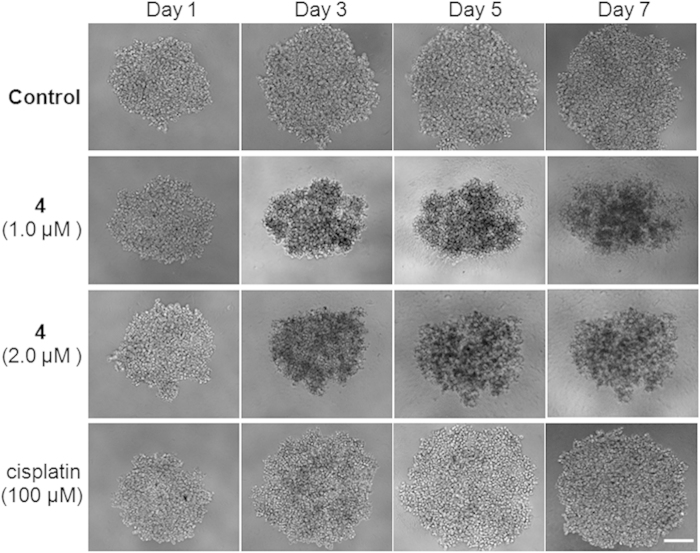
Growth inhibition of drug-treated A549R MCTSs. Scale bar: 200 μm.

Traditional research on the efficacy of cancer drugs is usually performed in two-dimensional (2-D) cell cultures, which may not be a true indicator of the *in vivo* effectiveness of cancer treatments. This is in part due to the absence of cell-cell or cell-extracellular matrix interactions in 2-D cell models^38^. Therefore, the development of cell culture models that can close the gap between conventional 2-D cell experiments and animal studies is necessary to study the cytotoxicity and anti-proliferative activities of drugs. Multicellular tumor spheroids (MCTSs) are heterogeneous cellular aggregates that have properties of solid tumors^39^,^40^, such as nutrient and oxygen gradients, hypoxic/necrotic regions, cell-cell matrix interactions, and gene expression. Hence, a 3D model, or MCTS, was introduced to mimic solid tumor *in vitro* and to study the viability of A549R cancer cells treated with the Ru(II) complexes. As shown in Table 1, the IC_50_ value of complex **4** on A549R MCTSs was 1.8 μM, which was 53-fold and 33-fold lower than complexes **2** and **3**, respectively, and far less than that of complex **1** and cisplatin. In addition, the sizes of the A549R MCTSs that were untreated or treated with cisplatin (100 μM) increased with time (Fig. 3), indicating that cisplatin was inactive against A549R cells. In contrast, the MCTSs treated with complex **4** (1.0 and 2.0 μM) decreased in size over time, and the MCTSs treated with complex **4** (1.0 μM) displayed distinct cell shrinkage, an increase in dark dead cells and a loss of extracellular matrix after 3 days. These observations demonstrated that complex **4** effectively inhibited A549R cell proliferation and killed A549R cancer cells at low concentrations.

### Cellular Uptake and Localization

**Figure 4:**
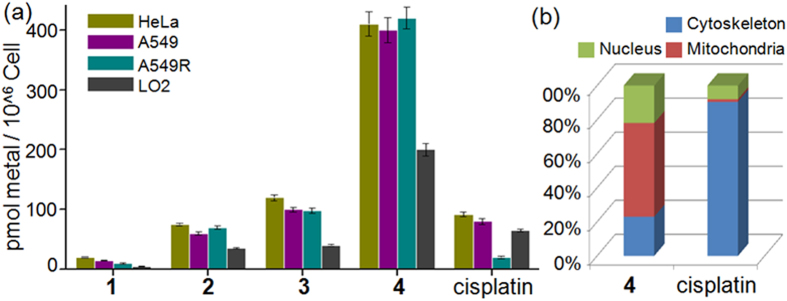
(**a**) The uptake of complexes in cell lines. (**b**) Subcellular distribution of complex **4** and cisplatin in A549R cells.

To understand the high sensitivity of cancer cells to Ru(II) complexes, the extent of cellular uptake was investigated with inductively coupled plasma mass spectrometry (ICP-MS) by treating the HeLa, A549, A549R and LO2 cell lines with 2.0 μM concentrations of the Ru(II) complexes and cisplatin for 12 h. As illustrated in Fig. 4a, significant differences were observed among the ruthenium complexes and cisplatin. In this study, the cellular uptake and lipophilicity were distinctly correlated and followed the order: **4** >**3** >**2** >**1**. Complex **4** had the best accumulation, but the amount of Ru in LO2 cells was far less than that in the three cancer lines, indicating selective accumulation and improved efficiency for treating cancer. Cisplatin was taken up more efficiently by the sensitive cell line (A549) than by the cisplatin-resistant cell line (A549R). The platinum level in A549 cells was ca. 4-fold higher than in A549R cells, suggesting that resistance to cisplatin was based on reduced uptake and enhanced efflux in A549R cells. Moreover, for the ruthenium complexes, similar concentrations of the metal were detected in HeLa and A549 cells compared to the A549R cells, indicating that the efflux system of these cells could not eliminate the Ru(II) complexes at these concentrations. Approximately 8% of complex **4** was taken up by the A549R cells, but less than 1% of platinum was accumulated in the A549R cells at the same concentration, suggesting that the high anticancer activity of complex **4** was attributed to accumulation. Moreover, over 55% of complex **4** was accumulated in the mitochondrial fraction of the A549R cells (Fig. 4b), which suggested that the mitochondria were the main targets of complex **4**. However, less than 1.5% of the cellular platinum was accumulated in the mitochondrial fraction of the A549R cells. Therefore, complex **4** could function in a different manner than cisplatin in A549R cells, which would be an appropriate solution to combat cisplatin resistance in tumor cells.

### Thioredoxin Reductase Inhibition

**Figure 5:**
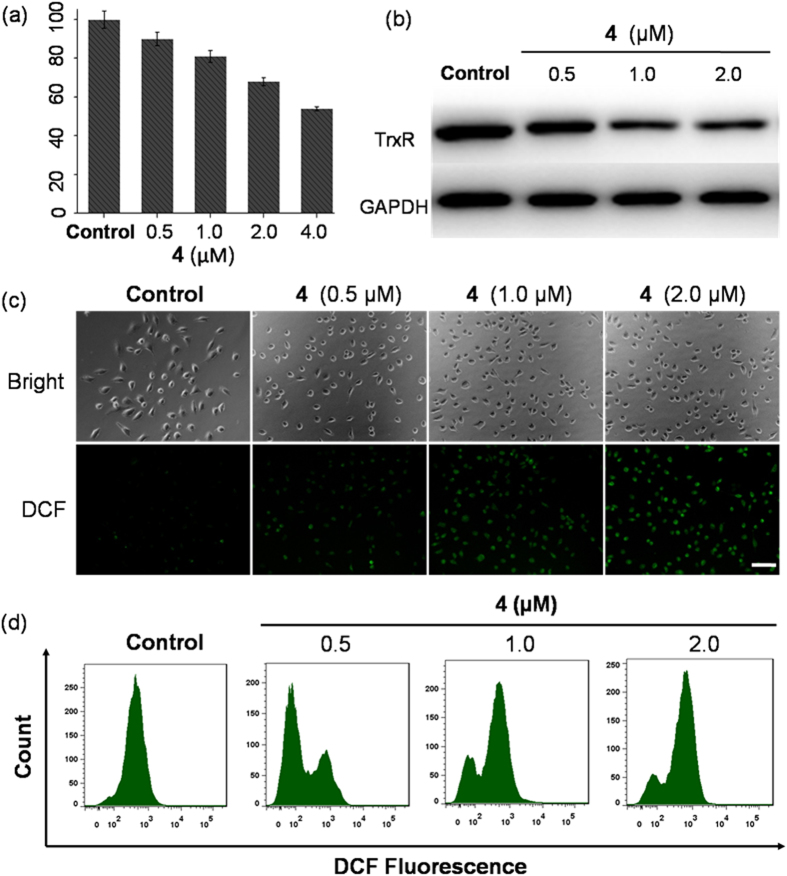
(**a**) Down-regulation of TrxR activity in A549R cells by complex **4**. (**b**) The expression levels of TrxR in complex **4**-treated A549R cells. (**c**) A549R cells showing an increase in ROS levels when treated with complex **4** under a fluorescence microscope. Scale bar: 100 μm. (**d**) The intracellular accumulation of ROS was detected by a DCHF-DA assay using flow cytometric analysis.

The elevated levels of TrxR in cancers, especially in non-small cell lung cancers, are responsible for drug resistance^41^. The thioredoxin system has a significant influence on intracellular physiological processes, such as antioxidant defense, cell functions, cell apoptosis and cell proliferation. TrxR has previously been identified as a drug target^42^. Therefore, the activity of TrxR in A549R cells treated with **4**, the most potent member of the series, was tested to confirm whether the TrxR inhibitory activity of complex **4** in A549R cells was responsible for the increased susceptibility of A549R cells. As illustrated in Fig. 5a, the TrxR activity of A549R cells was inhibited by treatment with **4** in a dose-dependent manner. At a concentration of 4.0 μM, intracellular TrxR activity declined by approximately 45%, which suggested that **4** directly inhibited intracellular TrxR in A549R cells. In addition, the expression levels of TrxR in A549R cells in response to the addition of complex **4** were also examined by Western blot analysis. As shown in Fig. 5b, the expression levels of TrxR were downregulated after treatment with different concentrations of complex **4**. Taken together, complex **4** exhibited strong inhibition of TrxR by decreasing its activity and protein levels, which may have contributed to the excellent cytotoxicity of complex **4** against A549R cells.

### Intracellular reactive oxygen species

TrxR is a key enzyme in the regulation of the intracellular redox environment^43^. However, cellular oxidative stress is known to trigger the initiation of apoptotic signaling, which results in cell death^44^,^45^. Complex **4** was proven to inhibit the activity of TrxR and could induce severe oxidative stress in A549R cells. To assess the capacity of complex **4** to generate intracellular reactive oxygen species (ROS), A549R cells were treated with different concentrations of complex **4** for 12 h and stained with 2′,7′-dichlorofluorescein diacetate (DCFH-DA)^46^. DCFH-DA is an oxidation-sensitive dye that is specifically detected by measuring the enhanced green fluorescent intensity caused by intracellular ROS. This dye was used to quantify the generation of intracellular ROS. As revealed in Fig. 5c, complex **4** had high expression levels in comparison to the ROS levels of the untreated controls. This revealed that an increase in ROS was observed by DCFH-DA staining when A549R cells were treated with 0.5 μM complex **4** for 12 h, and this increase was generated in a significant dose-dependent manner; these observations suggested that complex **4** could increase ROS levels in A549R cells. These findings were further confirmed with flow cytometric analyses (Fig. 5d).

### Mitochondrial Dysfunction

**Figure 6:**
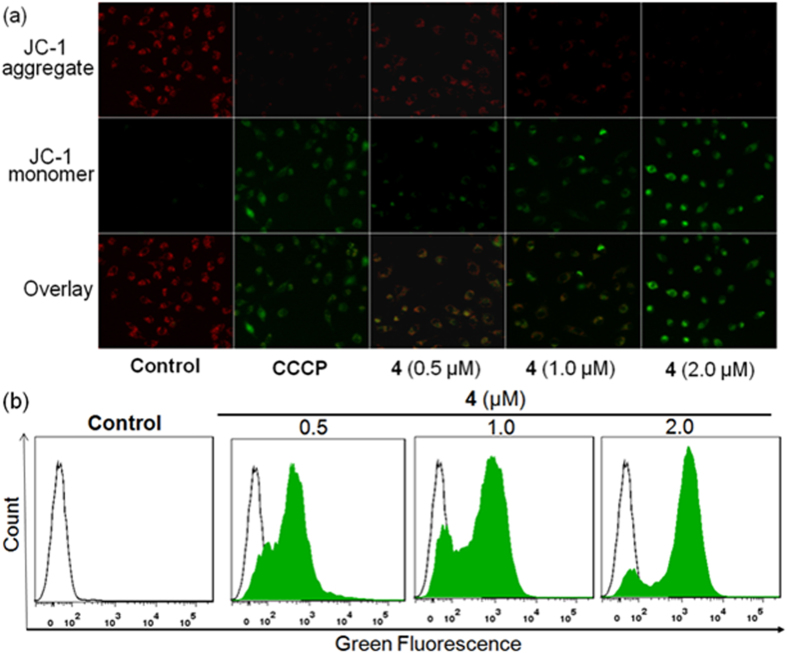
Changes of MMP in A549R cells treated with complex **4** with a JC-1 staining kit were observed under a fluorescence microscope (**a**) and analyzed using flow cytometry (**b**).

Mitochondria are the principal organelles related to energy metabolism and ATP synthesis; consequently, resistance against chemotherapeutic drugs can often be traced to the mitochondria^47^. Cancer cells exhibit many adaptive responses to drugs, including the optimization of mitochondrial function^48^. Mitochondrial dysfunction is involved in apoptotic cell death, and the loss of mitochondrial membrane potential (MMP, Δ*Ψ*_m_) is a hallmark of mitochondrial dysfunction^49^. We found that the mitochondria were the main targets of complex **4** in A945R cells. Confocal microscopy and flow cytometry were used to confirm whether complex **4**-induced apoptosis occurred by damaging mitochondria, and the cationic dye JC-1 was used as the MMP-sensitive probe^50^. As shown in Fig. 6a, A549R cells displayed a distinct red to green color shift in the presence of different concentrations of complex **4**, indicating the loss of MMP compared with the untreated group. The representative JC-1 green signals recorded by flow cytometry are exhibited in Fig. 6b. These results indicated that mitochondria-mediated pathways participated in the apoptosis of A549R cells caused by complex **4**.

### Cell cycle assay

Cell proliferation is activated under pathological conditions and plays a crucial role in cancer cell regeneration. Deregulated cell-cycle control is a fundamental aspect of cancer and the process is related to the proliferation and death of cancer cells^51^. Therefore, the effect of cyclometalated complex **4** on cell cycles was investigated using fluorescence-activated cell sorting (FACS) analysis of the DNA content. As shown in Figure S9, upon exposure of the A549R cells to complex **4**, the percentage of cells in the G0/G1 phase of the cell cycle decreased from 68.46 to 30.87. A higher dose of complex **4** (2.0 μM) elicited a strong G2/M arrest of A549R cells, accounting for 46% of the cell population (control, 12.82%). The enrichment of the G2/M phase of the cell cycle of A549R cells may have led to their apoptosis by disrupting the cell cycle.

### Cellular Apoptosis

**Figure 7:**
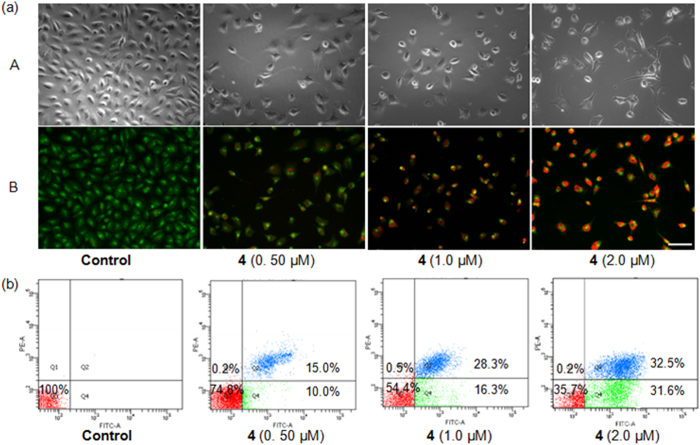
(**a**) Complex **4**-treated A549R cells were stained with AO/EB and observed under a fluorescence microscope. Scale bar: 100 μm. (**b**) Complex **4** induced apoptotic A549R cell death as examined by the Annexin V-FITC/PI assay.

Cell apoptosis is a naturally occurring, programmed and targeted cellular death mechanism that is very complex and is regulated by some proteins. This process is also greatly affected by extrinsic conditions^52^. Therefore, it is important to study the influence of drug-induced cellular apoptosis. Firstly, the induction of A549R cell apoptosis by complex **4** was investigated by apoptosis assays using AO/EB staining and fluorescence microscopy^53^. AO is a vital dye that can stain both live and dead cells and shows green fluorescence. EB only stains cells that have lost their membrane integrity and exhibits red fluorescence. Necrotic cells are stained in red but have nuclear morphologies that resemble those of viable cells. Apoptotic cells appear green and exhibit morphological changes, such as cell blebbing and the formation of apoptotic bodies. As shown in Fig. 7a, untreated A549R cells showed consistently green fluorescence with normal morphologies; however, the A549R cells treated with complex **4** (0.50–2.0 μM) showed orange fluorescence with fragmented chromatin and apoptotic bodies under a fluorescence microscope, suggesting that low concentrations of complex **4** predominantly induced apoptosis in A549R cells.

Soon after initiating apoptosis, the membrane phosphatidylserine (PS) is translocated from the inner to the outer leaflet of the plasma membrane. Therefore, PS can be easily detected by staining with fluorescently conjugated Annexin V, a 35–36 kDa Ca^2+^ dependent phospholipid-binding protein that has a high affinity for PS^54^. Cells that have bound Annexin V-FITC are stained green in the plasma membrane. Since the externalization of PS occurs in the earlier stages of apoptosis, FITC Annexin V staining is able to recognize apoptosis at an earlier stage compared to experiments that rely on nuclear changes such as DNA fragmentation^55^. Cells that have lost their membrane integrity show red staining (PI) throughout the nucleus and a halo of green staining (FITC) on the cell surface (plasma membrane). Therefore, flow cytometry analyses are able to distinguish between at least three different cell types during apoptosis: viable cells (Annexin V^-^ and PI^-^ negative), early apoptotic cells (Annexin V^+^ positive, but PI^−^ negative), and necrotic or late apoptotic cells (Annexin V^+^ and PI^+^ positive). A double-staining Annexin V/PI apoptosis kit and flow cytometry were used to investigate the nature of A549R cell death induced by complex **4**. The results are shown in Fig. 7b. At a concentration as low as 0.5 μM, 8.5% of the A549R cancer cells were in the early apoptotic phase after 24 h. At 1.0 and 2.0 μM, the number of cells at the early and late apoptotic phases greatly increased, indicating that complex **4** potently induced cell apoptosis.

## Discussion

Complex **1** is a known mitochondria-targeting anticancer drug candidate. Although this complex had an IC_50_ value higher than 100 μM, it did not show impressive cytotoxicity against cancer cell lines mainly due to its poor intracellular uptake, which limited its further application. Recently, Barton and Sadler explored the cellular cytotoxicity of complexes and found that the most lipophilic complexes exhibited the best cytotoxicity^56^,^57^. In addition, Pfeffer and our group have found that cyclometalation can greatly increase the anticancer activity of complexes by increasing their cellular uptake^58^. Although these four Ru(II) complexes possess very similar structures, they have distinct anticancer activities. Obviously, the lipophilicity of these Ru(II) complexes was regulated by altering the structures of the main ligands. In this study, the lipophilicity, cancer cell toxicity, and cell uptake were distinctly correlated, and followed the order of **3** >**2** >**1**. In addition, the cyclometalation made complex **4** the most lipophilic of all the complexes, which resulted in the highest cellular uptake and cytotoxicity. On the other hand, fluorine substitution in drug design has become commonplace. The special nature of fluorine is reported to impart a variety of properties to certain medicinal molecules, including enhanced protein-ligand binding interactions, adsorption, distribution, metabolic stability, changes in physicochemical properties, and selective reactivities. In this study, we found that the introduction of trifluoromethyl groups greatly increased the lipophilicity, cellular uptake and anticancer activities of ruthenium complexes. The cyclometalated complex **4** exhibited the strongest anticancer activity against cancer cells, and especially cisplatin-resistant tumor cells.

Drug resistance in the treatment of cancer and drug discovery is still a major obstacle. Although cisplatin is an effective anticancer agent for the treatment of several tumors, the occurrence of cisplatin resistance represents a serious clinical problem. The cellular mechanisms leading to cisplatin resistance are still not fully understood; however, decreased cellular concentration of cisplatin and increased rates of DNA repair are thought to be the main causes for cisplatin resistance. Consistent with previously reported data, complex **4** showed the most cellular uptake compared to the three other ruthenium complexes and cisplatin in A549R cells, and supplied sufficient drug concentrations to kill the A549R cells. Interestingly, as shown in Fig. 4b, the mitochondria of A549R cells were the main target of complex **4**, which suggests that complex **4** functions in a manner different from that of cisplatin. Thus, the novel mechanism of action and non-cross-resistance could allow complex **4** to overcome cisplatin resistance and show high cytotoxicity against A549R cells^59^. In addition, as a key enzyme in the regulation of the intracellular redox environment, TrxR plays a critical role in cancer progression and apoptosis. Complex **4** was found to increase ROS levels by inhibiting TrxR activity in A549R cells, which synergistically enhanced its anticancer activities.

In summary, our study revealed that the hydrophobicity and cellular uptake properties of these Ru(II) complexes were consistent with their cytotoxicity. Complex **4** was the most potent member of the series and accumulated well in A549R cancer cells. The complex also exhibited cytotoxic potency over 2 orders of magnitude higher than cisplatin in two-dimensional A549R cancer cells and 3D multicellular A549R tumor spheroids. Further studies demonstrated that complex **4** preferentially accumulated in the mitochondria of A549R cells and induced A549R cell apoptosis through multiple pathways including TrxR inhibition, intracellular elevated ROS levels, mitochondrial damage and cell cycle arrest. Our results also demonstrated that complex **4** is a potential anticancer candidate to overcome cisplatin resistance.

## Methods

### General Procedures

All reagents were purchased from commercial sources in reagent grade and were used as received, unless stated otherwise. The purity of the final Ru(II) complex was >95% as measured by HPLC. ^1^H NMR spectra were recorded on a Varian Mercury Plus 400 Nuclear Magnetic Resonance Spectrometer at 25 °C. Electrospray ionization mass spectra (ESI-MS) were recorded on an LCQ system (Finnigan MAT, USA), and the quoted/z-values given in this work are for the major peaks in the isotope distribution. Microanalyses (C, H, and N) were carried out using an Elemental Vario EL CHNS analyzer (Germany). All the tested complexes were dissolved in DMSO, and the concentration of DMSO was 1% (v/v). PBS solutions of complexes **1**–**4** were proven to be stable for at least 48 h at room temperature as monitored by UV/Vis spectroscopy before the experiments. Ru (III) chloride hydrate, 5′,6,6′-tetrachloro-1,1′,3,3′-tetraethylbenzimidazolyl-carbocyanine iodide (JC-1), carbonyl cyanide m-chloro-phenyl hydrazone (CCCP), propidium iodide (PI), cisplatin and 3-(4,5-dimethylthiazol-2-yl)-2,5-diphenyltetrazolium bromide (MTT) were obtained from Sigma-Aldrich. Benzo[h]quinoline was purchased from Alfa Aesar. The Annexin V-FITC apoptosis assay kit was purchased from Life Technologies, and the reactive oxygen species assay kit was obtained from the Beyotime Institute of Biotechnology (China). The thioredoxin reductase activity assay kit was purchased from Biovision.

### Purity Analyses

The purity of complexes **3** and **4** were determined using an Agilent Technology 1260 Infinity HPLC system equipped with a reverse-phase C18 column (APursuit XRs, 10 μm, 21.2 mm × 250 mm) operating at 30 °C. Elution was carried out using 0.1% (v/v) trifluoroacetic acid as mobile phase A and methanol as mobile phase B at a flow rate of 5.0 mL/min (10–90% aqueous MeOH with 0.1% formic acid over 20 min and MeOH with 0.1% formic acid from 20 to 30 min). Peaks were detected at 310 nm.

### Synthesis of 1-(4-tert-butylphenyl)-2-(4-(trifluoromethyl)phenyl)imidazo[4,5-f][1,10]phenanthroline (tbtfpip)

A mixture of 1,10-phenanthroline-5,6-dione (0.25 mmol), 4-tert-butylaniline (0.25 mmol), 4-(trifluoromethyl)benzaldehyde (0.25 mmol) and ammonium acetate (2.0 mmol) in glacial acetic acid (30 mL) was refluxed under argon for 12 h to obtain a pale-yellow solid. The solid was purified by column chromatography using dichloromethane–ethyl acetate (9:1) as the eluent. Yield: 100 mg, 80%. Anal. ESI-MS (m/z). 496.5 (M)^+^. Calcd for C_30_H_23_F_3_N_4_ (%): C, 72.55; H, 4.64; N, 11.29. Found (%): C, 72.71; H, 4.86; N, 11.55. ^1^H NMR (400 MHz, CDC_l3_) δ 9.22 (1 H, d, J = 3.6), 9.14 (1 H, d, J = 8.0), 9.07 (1 H, d, J = 3.9), 7.80–7.71 (3 H, m), 7.68 (2 H, d, J = 8.2), 7.57 (2 H, d, J = 8.2), 7.46 (3 H, d, J = 8.4), 7.31 (1 H, dd, J = 8.3, 4.3), 1.47 (9 H, s). HPLC purity: >95%.

### Synthesis of Ru(phen)_2_(tbtfpip)(PF_6_)_2_ (3)

This complex was synthesized with Ru(phen_2_)Cl_2_·2H_2_O (0.20 mmol) and **tbtfpip** ligand (0.20 mmol) in a 75% aqueous ethanol solution in one step, generating the product as a red powder. Yield: 187 mg, 75%. Anal. ESI-MS (m/z). 478.6 (M - 2PF_6_)^2+^. Calcd for C_54_H_39_F_15_N_8_P_2_Ru (%): C, 51.92; H, 3.13; N, 8.97. Found (%): C, 52.04; H, 3.36; N, 9.14. ^1^H NMR (400 MHz, DMSO) δ 9.20 (d, J = 9.1 Hz, 1 H), 8.83–8.73 (m, 4 H), 8.40 (d, J = 8.8 Hz, 4 H), 8.15–8.09 (m, 3 H), 8.06 (t, J = 4.7 Hz, 2 H), 8.00 (d, J = 4.6 Hz, 1 H), 7.91–7.86 (m, 1 H), 7.82 (d, J = 6.3 Hz, 5 H), 7.78 (dd, J = 13.2, 6.8 Hz, 5 H), 7.72 (s, 2 H), 7.56–7.50 (m, 1 H), 7.43 (d, J = 8.7 Hz, 1 H), 1.40 (s, 9 H). HPLC purity: >95%.

### Synthesis of Ru(phen)(bzq)(tbtfpip)(PF_6_) (4)

Benzo[h]quinoline (0.50 mmol) and η^6^-benzene (0.50 mmol) were added into 15 mL of MeCN, and the mixture was stirred for 24 h at 45 °C. Upon an anion exchange with NH_4_PF_6_, complex **4a** was obtained as a yellow solid. Complex **4a** and 1,10-phenanthroline (0.50 mmol) were then stirred for 12 h at room temperature, and the complex **4b** was produced. Complex **4b** was then subjected to photochemical conditions in acetone or MeCN under a 5.5 W UV lamp at room temperature for 6 h. The isomer of **4b** was produced and purified by column chromatography using dichloromethane-acetonitrile (9 : 1) as the eluent. Lastly, the ligand **L** (0.50 mmol) and the isomer **4b** were refluxed in methanol (15 mL) for 12 h, the solvent was evaporated under vacuum, the dark residue was dissolved in 5 mL of dichloromethane, and the solution was filtered through aluminum trioxide using a 9/1 CH_2_Cl_2_/MeCN eluent. The purple fraction was collected and evaporated to dryness. (Yield: 165 mg, 30%). Anal. ESI-MS (m/z). 957 (M - PF_6_)^+^. Calcd for C_55_H_39_F_9_N_8_PRu (%): C, 59.89; H, 3.54; N, 8.89. Found (%): C, 60.01; H, 3.85; N, 8.78. ^1^H NMR (400 MHz, DMSO) δ 8.56 (d, *J* = 7.4 Hz, 1 H), 8.51 (d, *J* = 8.1 Hz, 1 H), 8.31 (dd, *J* = 12.2, 6.0 Hz, 2 H), 8.25 (d, *J* = 1.4 Hz, 2 H), 8.18 (d, *J* = 4.4 Hz, 1 H), 8.08 (d, *J* = 5.2 Hz, 1 H), 8.03 (d, *J* = 4.5 Hz, 1 H), 7.96 (dd, *J* = 8.2, 5.1 Hz, 1 H), 7.88 (d, *J* = 8.8 Hz, 1 H), 7.87–7.76 (m, 7 H), 7.73 (d, *J* = 6.6 Hz, 3 H), 7.70 (s, 1 H), 7.68–7.63 (m, 2 H), 7.37 (dd, *J* = 7.8, 4.8 Hz, 3 H), 7.22 (d, *J* = 7.6 Hz, 1 H), 7.10 (t, *J* = 7.5 Hz, 1 H), 6.44 (d, *J* = 6.8 Hz, 1 H), 1.38 (s, 9 H). HPLC purity: >95%.

### Distribution coefficient studies

Distribution coefficients (log *P*_o/w_) were determined by liquid-liquid extraction between n-octanol (oil) and phosphate buffers (0.2 M, pH 7.4) with the flask-shaking method. N-Octanol and the buffer were mutually saturated with each other for at least 24 h before use. An aliquot of a ruthenium complex stock solution was added to the phosphate buffer concentrations at <10 mg/mL, and the mixture was shaken at 100 rpm for 24 h at 37 °C to allow for adequate partitioning. After the sample was centrifuged at 3000 rpm for 20 min, the aqueous layer was used for ruthenium analysis, and the Ru content in the aqueous layer was measured by ICP-MS and used to calculate the log *P*_o/w._

### Cell line and culture conditions

The human cervix carcinoma cell line (HeLa), the lung carcinoma cell lines (A549 and A549R), and the human hepatocyte cell line (LO2) were obtained from the Experimental Animal Center at Sun Yat-Sen University (Guangzhou, China). The cells were maintained in Dulbecco’s modified Eagle’s medium (DMEM, Gibco BRL) or Roswell Park Memorial Institute 1640 (RPMI 1640, Gibco BRL) medium supplemented with 10% fetal bovine serum (FBS, Gibco BRL), 100 μg/mL streptomycin, and 100 U/mL penicillin (Gibco BRL) in a humidified incubator under 5% CO_2_ and 20% O_2_ at 37 °C. To maintain the resistant phenotype, A549R cells were incubated with 5 μM cisplatin and cultured in drug-free medium for at least a week before use.

### Generation and analysis of MCTSs

MCTSs were cultured using the liquid overlay method. A549R cells in the exponential growth phase were dissociated by a trypsin/EDTA solution to gain single-cell suspensions. A number of 3000 diluted A549R cells were transferred to 1.5% agarose-coated transparent 96-well plates with 200 μL of DMEM containing 10% serum. The single cells formed MCTS aggregates approximately 400 μm in diameter after three days with 5% CO_2_ and 20% O_2_ at 37 °C. After formation of the MCTSs, each MCTS in a 96-well plate was imaged with a phase contrast microscope (Zeiss Axio Observer D1, Germany) using 10× objective to monitor their color, integrity, diameter, and volume.

### Cytotoxicity assay

The cytotoxicity of the ruthenium complexes and cisplatin were determined by an MTT assay. Briefly, cells were seeded into 96-well microtiter plates at 1 × 10^4^ cells per well and grown overnight at 37 °C in a 5% CO_2_ incubator; then, different concentrations of the complexes in cell culture medium were added to the cultures. The plates were then incubated in an incubator for 48 h under the same conditions. The stock MTT dye solution (15 μL, 5 mg/mL) was added to each well. After 4 h of incubation, the cultures were removed and 150 μL of DMSO was added to each well. The optical density of each well was measured on a microplate spectrophotometer at a wavelength of 595 nm.

### Cytotoxicity test on MCTSs

A549R MCTSs (diameters ≈400 μm) were treated by carefully replacing 50% of the medium with drug-supplemented standard medium using an eight-channel pipet. In parallel, 50% of the medium containing solvent was replaced with solvent-free medium for the untreated MCTSs. Three A549R MCTSs were treated per condition and drug concentration; the DMSO volume was less than 1% (v/v). The MCTSs were then allowed to incubate for another 48 h. The cytotoxicities of the Ru(II) complexes toward the MCTSs were measured using ATP concentrations with a Cell TiterGlo kit (Promega). After 30 min of incubation, the MCTSs were carefully transferred into black-sided, flat-bottomed 96-well plates (Corning) and mixed with a pipet for luminescence measurements on an Infinite M200 PRO instrument (TECAN).

### Cell uptake studies

The cells were plated at a density of 1 × 10^5 ^cells/mL in 10 mL of DMEM for 24 h at 37 °C under 5% CO_2_ and 20% O_2_, Ruthenium complexes (2.0 μM) and cisplatin were added to the culture medium and incubated for 12 h, and mixed with cold PBS and a trypsin-EDTA solution in a centrifuge tube for counting. Then, the nuclei and cytoplasm of the cells were extracted by using a Nuclear and Cytoplasmic Protein Extraction Kit (Life Technologies). The samples were digested in 20% HNO_3_ and 10% H_2_O_2_ at room temperature overnight. Each sample was then diluted with Milli-Q water to obtain 2% HNO_3_ sample solutions. Ruthenium contents were determined using inductively coupled plasma mass spectrometry (ICP-MS Thermo Elemental Co., Ltd.).

### Real-time cell growth and proliferation assay

This experiment was performed using an xCELLigence RTCA DP system real time cell analyzer (Roche Diagnostics GmbH, Germany). In brief, 80 μL of cell culture media was added to each well of a 16-well plate (E-plate 16, Roche Diagnostics GmbH, Germany). The 16-well plates were then connected to the system and checked for suitable electrical contacts in the real time cell analyzer, and the background impedance was measured for 24 s. Meanwhile, the A549R cells were suspended in cell culture medium and adjusted to 5 × 10^4 ^cells/mL, and 100 μL of each cell suspension was added to each well of 6-well plates. Approximately 24 h after seeding, the A549R cells were in the logarithmic phase of growth, and a wide range of concentrations of complex **4** and cisplatin (100 μM) were added. The cells were automatically monitored every 15 min over 48 h by the xCELLigence system. Data were analyzed using RTCA software (version 1.2) that was supplied with the instrument.

### Subcellular distribution studies

A549R cells were plated at a density of 1 × 10^5 ^cells/mL in 10 mL of DMEM for 24 h at 37 °C under 5% CO_2_ and 20% O_2_. Complex **4** (2.0 μM) and cisplatin (2.0 μM) were added to the culture medium and incubated for 12 h and then suspended in cold PBS and a trypsin-EDTA solution in a centrifuge tube for counting. Then, the nuclei and cytoplasm of the cells were extracted using a Nuclear and Cytoplasmic Protein Extraction Kit (Life Technologies). The samples were digested in 20% HNO_3_ and 10% H_2_O_2_ at room temperature overnight. Each sample was then diluted with Milli-Q water to obtain 2% HNO_3_ sample solutions. The ruthenium or platinum contents were determined using inductively coupled plasma mass spectrometry (ICP-MS Thermo Elemental Co., Ltd.).

### Measurement of intracellular reactive oxygen species

The generation of reactive oxygen species (ROS) in A549R cells was measured using a ROS sensitive fluorescent probe, 2,7-dichlorodihydrofluorescein diacetate (DCFH-DA). This probe can be oxidized to 2′,7′-dichlorofluorescein (DCF) by ROS and exhibits increased green fluorescence intensity. Briefly, the cultured A549R cells were treated with complex **4** at different concentrations (i.e., 0.5, 1.0, or 2.0 μM) and the untreated cells were maintained as the control. After incubation for 12 h, the cells were harvested, washed twice, resuspended in 10 mM DCFH-DA and incubated at 37 °C for 30 min in the dark. The levels of intracellular ROS were examined with an inverted fluorescence microscope (Zeiss Axio Observer D1) and flow cytometry (FACSCanto II, BD Biosciences, USA). The excitation wavelength was 485 nm, and the fluorescence was measured at 530 nm. Data acquisition and analyses were carried out using FlowJo software.

### Cell cycle analysis

A549R cells were treated with different concentrations of complex **4** (i.e., 0.5, 1.0 and 2.0 μM) for 24 h. The cells were collected and fixed in 2 mL of 70% cold aqueous ethanol (v/v). After incubation, the cells were centrifuged and washed three times with cold PBS and resuspended in 0.5 mL of PBS. To a 0.5 mL cell sample, 50 μM of RNase A (1 mg/mL in PBS) was added and incubated for 30 min at 37 °C; then, 50 μM of a PI (500 mg/mL in PBS) solution was added after gentle mixing for 5 min at 37 °C in the dark. The samples were resuspended and 10,000 cells were analyzed for each sample by a BD FACS Calibur cytometer (Becton Dickinson, Heidelberg, Germany). The data were acquired and analyzed with ModFit LT 3.2.

### Annexin V/PI double staining

A549R cells were added to a 6-well plate at 5 × 10^5^ cells per well with various concentrations (i.e., 0.5, 1.0 and 2.0 μM) of complex **4** and incubated for 24 h. Cells were centrifuged, washed twice with cold PBS, and resuspended in 0.5 mL of binding buffer from an Annexin V/PI apoptosis Kit (MultiSciences (Lianke) BiotechCo., Ltd.). Then, 5 μL of Annexin V-FITC and 10 μL of PI were added to the sample solution. After incubation for 5 minutes in the dark, the specimens were quantified by flow cytometry on a FACS Canto II (BD Biosciences, USA).

### Analysis of MMP

A549R cells were cultured in 6-well tissue culture plates for 24 h and treated with complex **4** (0.5, 1.0 and 2.0 μM) for 24 h. After the treatment, the cells were collected and resuspended at 1 × 10^6^ cells/mL in a pre-warmed staining working solution containing JC-1 (5 μg/mL) and incubated for 15 min at 37 °C. Subsequently, the cells were washed twice with pre-warmed PBS and analyzed immediately with a flow cytometer on a FACS Canto II (BD Biosciences, USA). Red and green mean fluorescence intensities were analyzed using FlowJo 7.6 software (Tree Star, OR, USA). 10,000 events were acquired for each sample. After the treatment described above, the stained cells were visualized under an inverted fluorescence microscope (Zeiss Axio Observer D1). JC-1 fluorescence was measured with single excitation (488 nm) and dual emission (shift from green 530 nm to red 590 nm).

### TrxR activity assay in A549R cells

A549R cells (2 × 10^6^ cells) treated or untreated with complex **4** for 12 h were homogenized in 100–200 μl of cold assay buffer on ice and a protease inhibitor cocktail was added to the sample; the sample was then centrifuged at 10,000 x g for 15 min at 4 °C. The supernatant was collected for the assay and stored on ice. The protein concentration of each supernatant was determined using the Bradford reagent. A total of 40 μl of the reaction mix (30 μl assay buffer, 8 μl 5, 5′-dithiobis (2-nitrobenzoic) acid (DTNB) solution and 2 μl NADPH) was added to each sample and incubated at 25 °C for 20 min. TrxR catalyzes the reduction of DTNB by NADPH to 5-thio-2-nitrobenzoic acid (TNB2), which generates a strong yellow color (λ_max_ = 412 nm); consequently, the amount of DTNB was used to monitor the change in absorbance at 412 nm. Two assays were performed: the first measurement was the total DTNB reduction by the sample, and the second was DTNB reduction by the sample in the presence of the TrxR-specific inhibitor. The difference between the two results was the reduction of DTNB by TrxR. The TrxR activity was calculated by subtracting the absorbance of the background control from the absorbance of the sample; these data were used to determine the percentage of TrxR activity in comparison with the control. The DTNB reduction assay was then used to measure intracellular enzyme activity; the intracellular enzyme activities are given as percentages of the control.

### Western blot analysis

A549R cells were seeded into 10 cm tissue culture dishes (Corning), incubated for 24 h, and treated with complex **4** for 12 h. The cells were washed with ice-cold PBS and lysed by incubation in radio immune precipitation assay buffer (RIPA) and a protease inhibitor cocktail (Sigma) for 30 min on ice. The lysates were centrifuged at 15000 rpm for 15 min at 4 °C, and the protein concentrations were quantified by a BCA protein assay reagent kit (Sigma). The proteins were separated on precast NuPAGE 4% to 12% polyacrylamide gradient Bis–Tris gels (Invitrogen) under denaturing conditions, transferred to PVDF membranes (Invitrogen), and subjected to Western blot analyses. Thioredoxin reductase antibody (Proteintech USA) and rabbit anti-GAPDH polyclonal antibody (Proteintech USA) were diluted (1:2000, respectively) in PBS containing 5% nonfat powdered milk and 0.1% Tween-20 and incubated with the membrane overnight at 4 °C. Horseradish peroxidase conjugated secondary antibodies (Proteintech USA) were used. The bound immune complexes were detected using an ECL prime Western blot detection reagent (Amersham Inc., USA). The images were captured on FluorChem M (ProteinSimple, Santa Clara, CA).

## Additional Information

**How to cite this article**: Zeng, L. *et al*. Ruthenium(II) Complexes with 2-Phenylimidazo[4,5-f][1,10]phenanthroline Derivatives that Strongly Combat Cisplatin-Resistant Tumor Cells. *Sci. Rep.* **6**, 19449; doi: 10.1038/srep19449 (2016).

## Supplementary Material

Supplementary Information

## Figures and Tables

**Figure 1:**
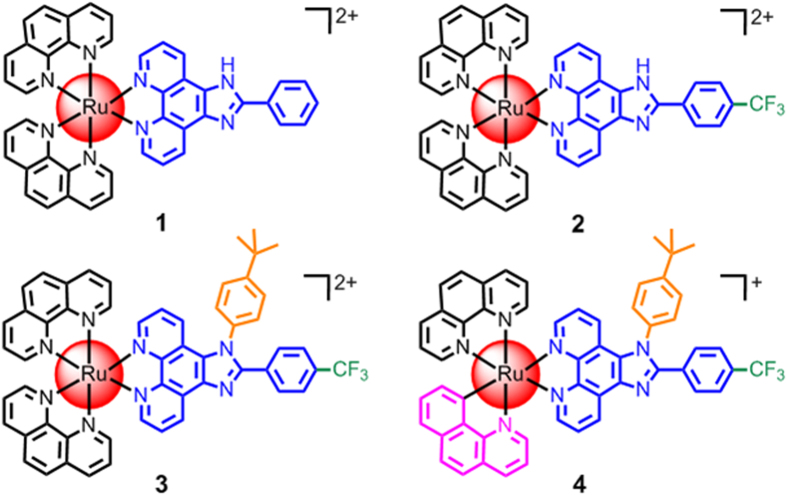
The chemical structures of Ru(II) complexes **1**–**4**.

**Table 1:** Cytotoxic activities of complexes 1–5 and their IC_50_ values (μM)^a^.

Complexes	1	2	3	4	cisplatin
HeLa	>100	55.4 ± 3.1	22.5 ± 3.3	2.4 ± 0.3	15.1 ± 2.1
A549	>100	43.4 ± 5.3	28.6 ± 2.6	1.0 ± 0.2	21.3 ± 3.3
A549R	>100	48.2 ± 2.7	32.2 ± 1.5	0.8 ± 0.1	142.5 ± 5.4
LO2	>100	75.8 ± 7.5	45.6 ± 3.8	6.7 ± 2.4	18.9 ± 2.6
MCTSs^b^	>100	95.7	60.6 ± 5.5	1.8 ± 0.3	>200
Resistant Index^c^	–	1.11	1.13	0.8	6.69

^a^Cells were treated with various concentrations of the complexes for 48 h.

^b^A549R MCTSs (~400 μM in diameter) were treated with various concentrations of the complexes for 48 h.

^c^IC_50_(A549R)/IC_50_(A549). Each value represents the mean ± SD of three independent experiments.
