# Supplementary material for: Ruthenium(II) Complexes with 2-Phenylimidazo[4,5-f][1,10]phenanthroline Derivatives that Strongly Combat Cisplatin-Resistant Tumor Cells
Source: Sci Rep. 2016 Jan 14;6:19449. doi: 10.1038/srep19449 (PMC4725915; doi:10.1038/srep19449)
Supplement: Supplementary Information [file srep19449-s1.doc]

Supporting Information

**Ruthenium(II) Complexes with 2-Phenylimidazo[4,5-f][1,10]phenanthroline Derivatives that Strongly Combat Cisplatin-Resistant Tumor Cells**

Leli Zeng, Yu Chen, Jiangping Liu, Huaiyi Huang, Ruilin Guan,

Liangnian Ji & Hui Chao*

*MOE Key Laboratory of Bioinorganic and Synthetic Chemistry, School of Chemistry and Chemical Engineering, Sun Yat-Sen University, Guangzhou 510275 China*

## *E-mail:* [*ceschh@mail.sysu.edu.cn*](mailto:ceschh@mail.sysu.edu.cn)

**Figure. S1** The synthetic protocols of ligand **L** and complex **4 S2**

**Figure. S2** ESI-MS spectra of ligand **L** in CH_3_OH solutions **S3**

**Figure. S3** ESI-MS spectra of complex **3** in CH_3_OH solutions **S3**

**Figure. S4** ESI-MS spectra of complex **4** in CH_3_OH solutions **S4**

**Figure. S5** ^1^H NMR spectra of **tbtfpip** in CDC_l3_ **S4**

**Figure. S6** ^1^H NMR spectra of complex **3** in DMSO-*d6* **S5**

**Figure. S7** ^1^H NMR spectra of complex **4** in DMSO-*d6* **S5**

**Figure. S8** Octanol/water partition coefficients of Ru(II) complexes **S6**

**Figure. S9** Effect of complex **4** on cell cycle populations **S6**


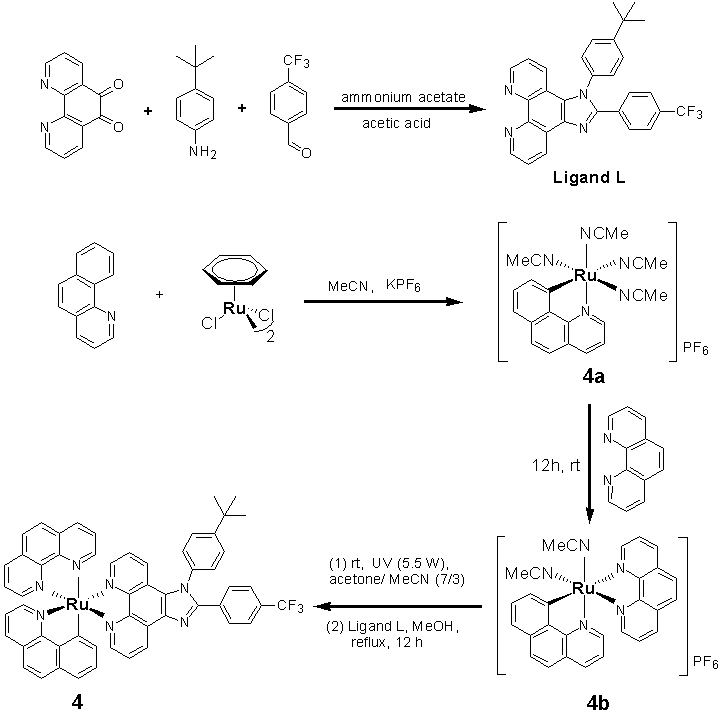


**Figure. S1** The synthetic protocols of ligand **L** and complex **4**.

**
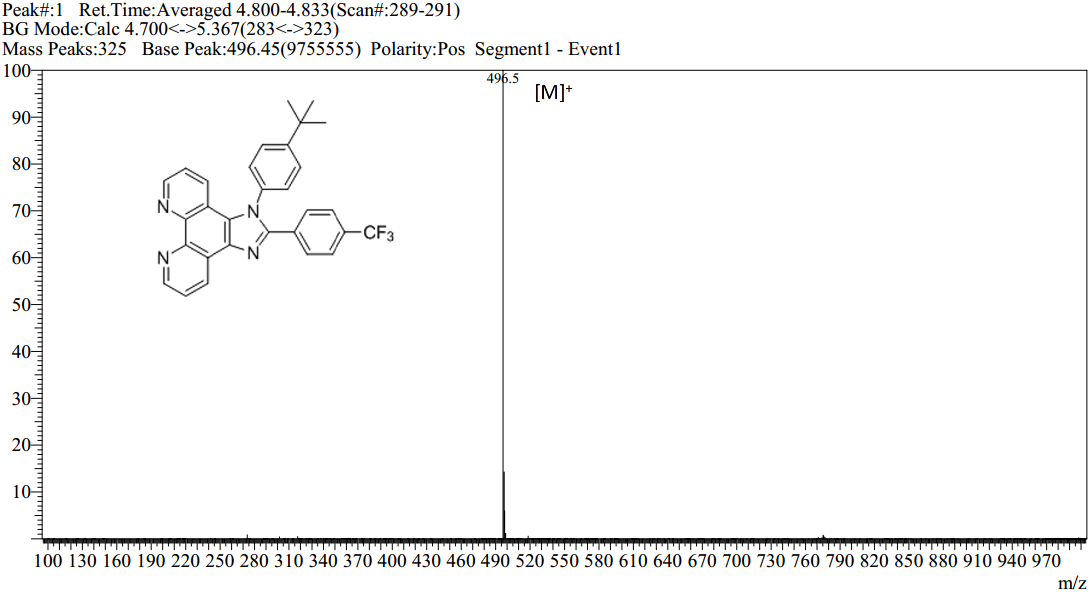
Figure. S2** ESI-MS spectra of ligand **L**.


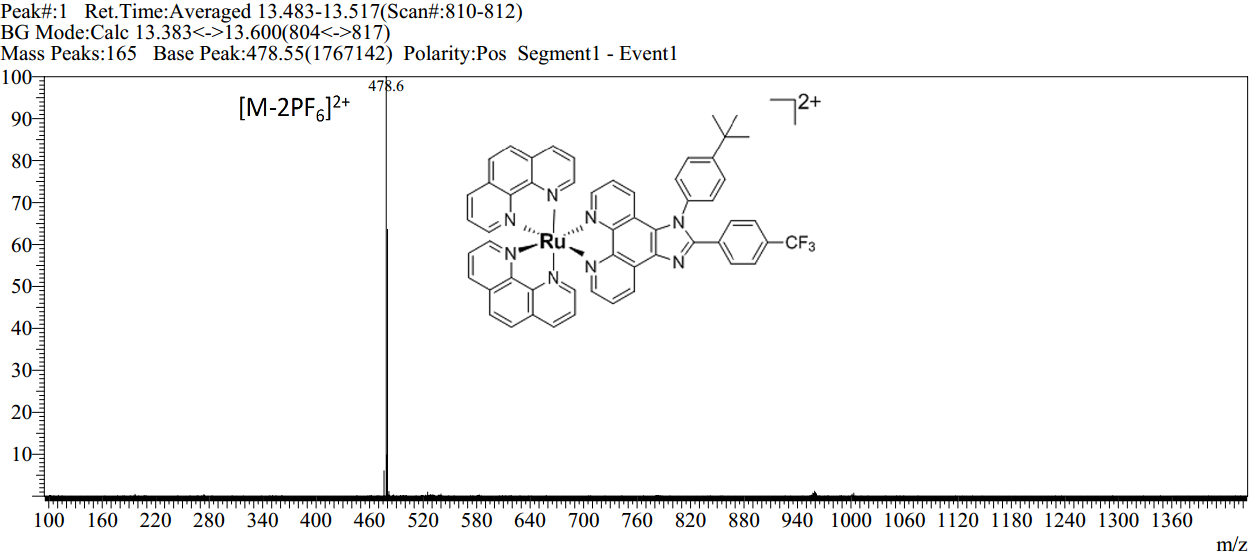


**Figure. S3** ESI-MS spectra of complex **3**.


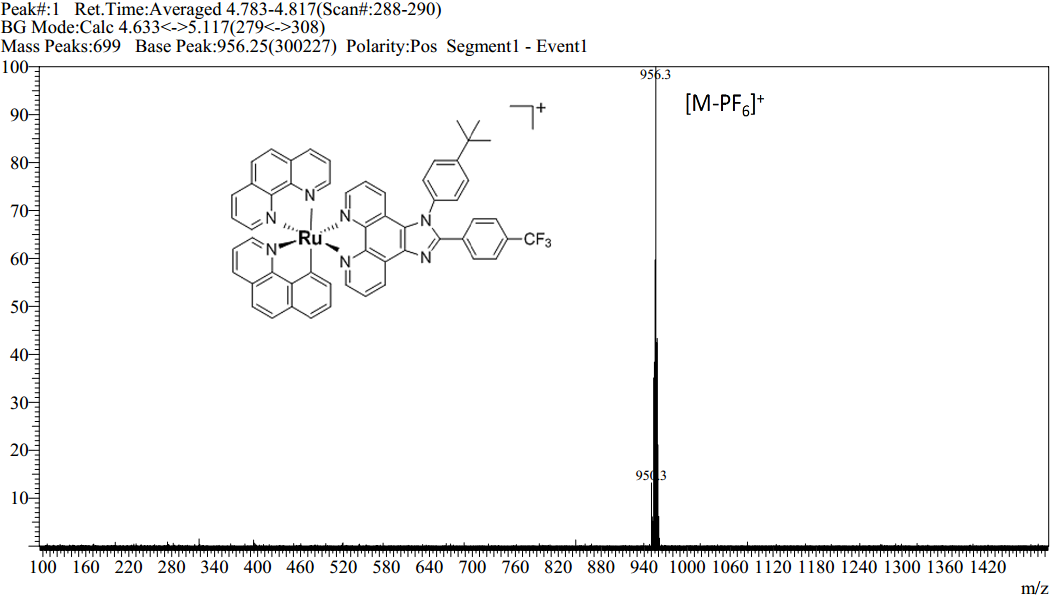


**Figure. S4** ESI-MS spectra of complex **4**.


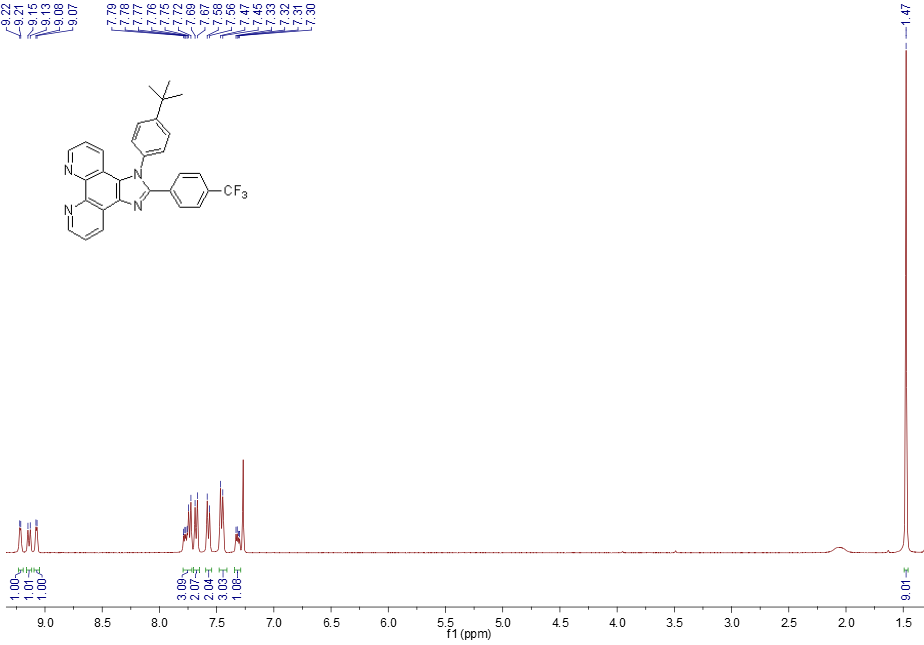


**Figure. S5** ^1^H NMR spectra of **tbtfpip**.

**
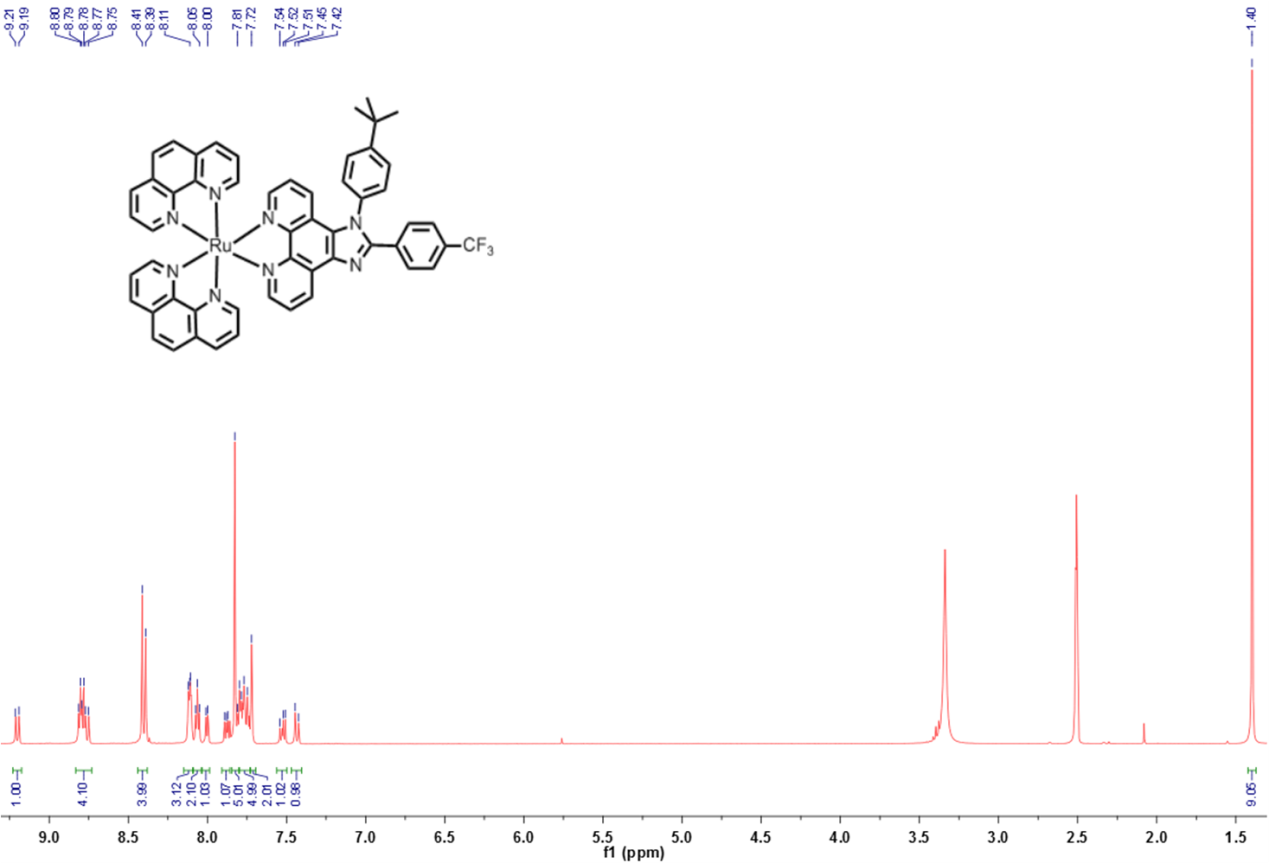
**

**Figure. S6** ^1^H NMR spectra of complex **3**.

**
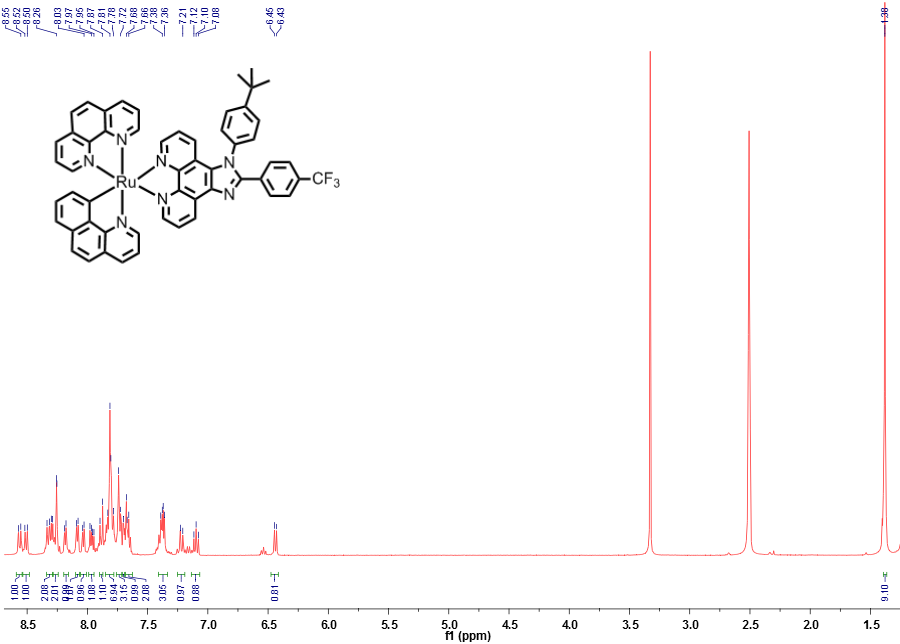
**

**Figure. S7** ^1^H NMR spectra of complex **4**.


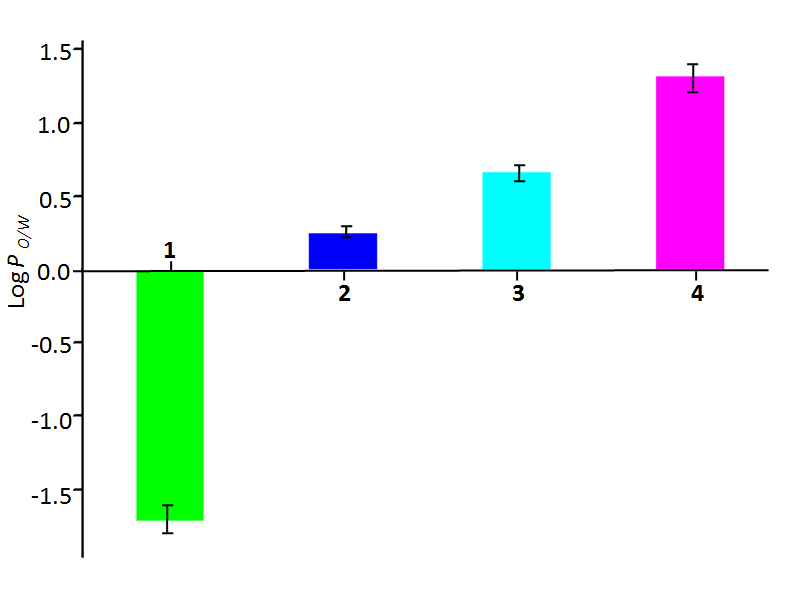


**Figure. S8** Octanol/water partition coefficients of Ru(II) complexes.


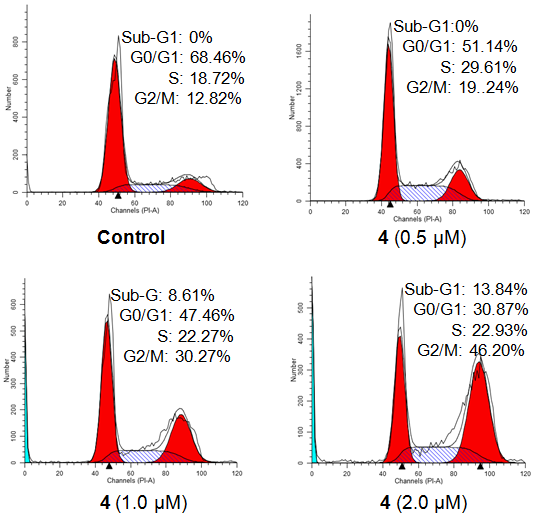


**Figure. S9** Effect of complex **4** on the distribution of A549R cells in cell cycle populations after 24 h of treatment.
